# Obtaining Cellulose Nanocrystals from Olive Tree Pruning Waste and Evaluation of Their Influence as a Reinforcement on Biocomposites

**DOI:** 10.3390/polym15214251

**Published:** 2023-10-28

**Authors:** Sofía Jurado-Contreras, Francisco J. Navas-Martos, Ángeles García-Ruiz, José A. Rodríguez-Liébana, M. Dolores La Rubia

**Affiliations:** 1 Andaltec Technological Centre, Ampliación Polígono Industrial Cañada de la Fuente, C/Vilches 34, Martos, 23600 Jaén, Spain; sofia.jurado@andaltec.org (S.J.-C.); francisco-javier.navas@andaltec.org (F.J.N.-M.); jose-antonio.rodriguez@andaltec.org (J.A.R.-L.); 2 Department of Chemical, Environmental and Materials Engineering, Campus Las Lagunillas, University of Jaén, 23071 Jaén, Spain; jogarcir@ujaen.es; 3 University Institute for Research in Olive Grove and Olive Oil (INUO), Campus Las Lagunillas, University of Jaén, 23071 Jaén, Spain

**Keywords:** polylactic acid, composites, nanocellulose, olive tree pruning

## Abstract

The objective of this work is to improve the mechanical properties of polylactic acid (PLA) by incorporating cellulose nanocrystals (CNC) previously obtained from a cellulose pulp extracted from olive tree pruning (OTP) waste. Composites were manufactured by melt processing and injection moulding to evaluate the effect of the introduction of CNC with conventional manufacturing methods. This OTP-cellulose pulp was subjected to a further purification process by bleaching, thus bringing the cellulose content up to 86.1%wt. This highly purified cellulose was hydrolysed with sulfuric acid to obtain CNCs with an average length of 267 nm and a degradation temperature of 300 °C. The CNCs obtained were used in different percentages (1, 3, and 5%wt.) as reinforcement in the manufacture of PLA-based composites. The effect of incorporating CNC into PLA matrix on the mechanical, water absorption, thermal, structural, and morphological properties was studied. Maximum tensile stress and Young’s modulus improved by 87 and 58%, respectively, by incorporating 3 and 5%wt. CNC. Charpy impact strength increased by 21% with 3%wt. These results were attributed to the good dispersion of CNCs in the matrix, which was corroborated by SEM images. Crystallinity index, glass transition, and melting temperatures were maintained.

## 1. Introduction

One of the main drawbacks of using petroleum-derived polymeric materials is the accumulation of waste derived from their use. The low biodegradability of these materials produces serious ecological imbalances and health risks [1,2]. In addition, these conventional plastics are non-renewable [3]. Currently, a large amount of research has been conducted to mitigate these problems in two main ways: (i) reuse and recycling of conventional plastics; (ii) manufacturing of new biodegradable polymeric materials (green composites) [2,4]. Biobased composites are hybrid materials that combine a polymeric matrix, from biological resources, with a suitable percentage of particles in order to obtain a material with improved properties. They have recently attracted increasing interest due to their good biocompatibility, low cost, density and toxicity, and biodegradability [5,6]. Among the most widely used biobased polymer matrices are starch, polylactic acid (PLA), and cellulose acetate [5,7].

PLA is a thermoplastic aliphatic polyester produced from renewable resources that has some advantages including high industrial availability, easy processing, and biodegradability [8,9]. Typical applications of PLA include plastic bags for bio-waste, disposable cups and plates, among others [9]. On the other hand, PLA has some serious disadvantages such as brittleness, low barrier properties, and poor heat resistance. Nevertheless, the addition of cellulosic fillers to the PLA matrix provides great advantages such as high crystallinity and thermal stability [10]. In addition, cellulose is an abundant renewable raw material whose use in plastics can contribute to reduce CO_2_ emissions and air pollution [11,12]. The amorphous domains of cellulose can be removed by chemical and physical treatments, resulting in crystals known as cellulose nanocrystals (CNC) [7]. CNCs are a type of biofiller that can be extracted from a variety of lignocellulosic wastes such as those from wood, sisal, cotton, kenaf, bamboo crops, hemp, flax, wheat straw, mulberry bark, ramie, Avicel, tunicin, cellulose from algae, and bacteria [13,14,15,16]. CNCs are identified as one of the most promising polymer reinforcements due to their high aspect ratio and the large amount of free -OH groups on their surface, which favours their biocompatibility [17]. In contrast, the hydrophilic nature of nanometer-sized cellulose makes it difficult to properly disperse it into hydrophobic polymer matrices, which implies a decrease in mechanical properties of the resulting bionanocomposite [8,18]. When used as reinforcement, the geometric structure of the CNCs and the percentage incorporated into the polymeric matrix are the most important factors that determine the strength and stiffness of the final nanocomposites [19]. The incorporation of CNC not only produces compounds with highly desirable properties, but also helps to lower the overall price of the resulting compounds. These materials are useful in a wide variety of applications due to the strict environmental legislations in force in several countries [20]. Possible applications include the automotive industry (door panels, boot linings, wheel housings, storage panels, etc.), packaging (food packaging, bottles), construction (door panels, frames, furniture, etc.) [21] and biomedical applications (injectable tissue scaffolds, bone tissue regeneration, vascular grafts, improved bone implant adhesion, and drug delivery) [22].

Olive crops cover 11.6 million hectares worldwide. The large amount of biomass generated as a consequence of the tasks related to the necessary pruning of the olive tree, generally carried out every two years, can be considered one of the most relevant renewable resources [23]. The disposal of these olive tree pruning (OTP) residues to keep the fields clean and prevent the spread of diseases is commonly carried out by burning in the own croplans, which increases air pollution [24]. OTP has an approximate composition by weight of 39% cellulose, 14% lignin, and 22% hemicellulose. The cellulose content of the OTP is higher than that reported by other authors for various wastes such as tea waste [25], sweet sorghum [26], rice husk [27], apple tree pruning, and pea stalks [28], among others. The composition of OTP makes it a suitable material for the production of several products such as microcrystalline cellulose and nanocellulose [29]. Ibarra et al. (2021) obtained microfibrillated cellulose from OTP by physical pre-treatment. The final material presented high crystallinity and had similar properties to those of microfibrillated cellulose obtained from eucalyptus pulp [30].

There are several research works that have studied the reinforcement of a PLA matrix with nanocellulose for food packaging by using lab-scale solvent casting methods [31,32,33]. However, not many studies have been found on the melt processing of PLA composites with nanocellulose, even though it is expected to be a key processing method to bring these materials to the high-volume product market, mainly due to its low cost [6]. Jonoobi et al. (2010) reinforced a PLA matrix with cellulose nanofibres (CNF), isolated from kenaf pulp using mechanical procedures, at 1, 3, and 5%wt. [8]. Eyholzer et al. (2012) reported an increase in mechanical properties by manufacturing CNF-reinforced PLA bionanocomposites [34]. Similarly, Okubo et al. (2009) studied the incorporation of CNF (1–2%wt.) obtained from bamboo as a reinforcing agent of a PLA matrix [35].

The novelty of this study is that it is focused on the use of a value-added product based on cellulosic biomass, obtained from OTP waste, as a reinforcement of PLA. Furthermore, this study presents an innovative and extensive characterisation in terms of morphological, mechanical, and thermal properties of the composites, which were manufactured by melt compounding and subsequently processed in an injection moulding machine to obtain the test specimens. The main objectives are as follows: (a) to increase the mechanical properties of a PLA matrix; (b) to expand the range of industrial applications of the material; (c) to make products manufactured with these composites more environmentally sustainable; and (d) to provide information to the scientific community for future potential applications.

## 2. Materials and Methods

### 2.1. Isolation of Cellulose Content

The OTP used as raw material for the synthesis of CNC was previously conditioned and treated as described in our previous work [36]. Briefly, OTP was firstly subjected to a conditioning process (grinding and sieving), and then to a two-step chemical treatment (acid + alkaline hydrolysis) to obtain a pulp enriched in cellulose labelled as OTP-BH. HNO_3_ (Puriss. p. a.,65–67%), NaOH pellets (≥99%) and H_2_O_2_, were purchased from DICSA S.L. (Spain). The acid treatment was carried out with HNO_3_ at 8% *v*/*v*, at 90 °C for 240 min. The resulting biomass, depleted in hemicellulose and lignin, was further hydrolysed by following an alkaline reaction with NaOH 6% *w*/*v*, at 75 °C for 105 min. Then, a bleaching treatment was also performed to remove the remaining non-cellulosic components (hemicellulose and lignin) and to further increase the cellulose content of the OTP-BH sample. The bleaching reaction was performed at 1:20 solid-liquid ratio with an aqueous 2% *v*/*v* H_2_O_2_ solution at 70 °C for 1 h. Before bleaching, the pH of the solution was adjusted to values of 11–12 by addition of NaOH. Finally, the resulting bleached sample (OTP-BL) was washed until neutral pH and dried at room temperature.

The yield of the bleaching treatment (*Yield_BL_*) was obtained by using Equation (1):

(1) $${Yield}_{BL}\;\left(\%\right)=\frac{m_{OTP-BL}}{m_{OTP-BH}}\cdot 100$$

where *m_OTP-BL_* and *m_OTP-BH_* are the dry weight masses of the fibres after and before bleaching, respectively.

To check the effect of the bleaching treatment, both OTP-BH and OTP-BL fibres were characterised in terms of moisture, ash, and chemical composition. The moisture and ash contents were determined using TAPPI T 12 os-75 and TAPPI T 15 os-58 methodologies, respectively. Cellulose, hemicellulose, and lignin content were calculated using the methodology proposed by Browning [37], with some modifications, in a High-Performance Liquid Chromatography (HPLC) system, as described in Rodriguez-Liébana et al. (2023) [36].

### 2.2. Preparation of CNCs

The synthesis of CNCs was carried out by acid hydrolysis with 64% *v*/*v* sulfuric acid, with a solid/liquid ratio of 1:20 at 40 °C with constant stirring for 40 min. At the end of the reaction time, cold ultrapure water (3 °C) was added to quench treatment. Afterwards, neutralization was carried out by successive centrifugation cycles of 15 min at 8000 rpm, until reaching pH values ≥ 6. Finally, a lyophilizer Module D (Thermo Savant, Western Ave Haverhill, MA, USA) was used to obtain the CNCs in solid form.

The calculation of the yield of the CNC (*Yield_CNC_*) synthesis process was carried out using Equation (2):

(2) $${Yield}_{CNC}\;\left(\%\right)=\frac{m_{CNC}}{m_{OTP-BL}}\cdot 100$$

where *m_CNC_* is the weight of nanocellulose obtained after the freeze-drying process and *m_OTP-BL_* is the initial weight of OTP-BL.

In order to calculate the overall yield of the CNC (*Yield_OTP-CNC_*) production from the raw residue OTP, Equation (3) was used:

(3) $${Yield}_{OTP-CNC}\;\left(\%\right)={(Yield}_{BH}\cdot {Yield}_{BL}\cdot {Yield}_{CNC})\cdot 100$$

where *Yield_BH_*, *Yield_BL,_* and *Yield_CNC_* are the yields of the first stage of cellulose purification, bleaching, and CNC production, respectively.

### 2.3. Characterization of OTP Samples and CNCs

Morphological characterization of CNC was performed by transition electron microscopy (TEM) using a transmission electron microscope JEM-1010 (JEOL, Tokyo, Japan) operating at a voltage of 80 kV. ImageJ v.153t software was used to determine the mean particle size and distribution, measuring approximately 50 nanocrystals.

The structural composition of the OTP-BH, OTP-BL, and CNC was determined by Fourier transform infrared spectroscopy (FT-IR) and X-ray diffraction (XRD). An FT-IR spectrometer Tensor 27 (Bruker, Billerica, MA, USA) was used for FT-IR analysis in the spectral range of 400–4000 cm^−1^ with a resolution of 4 cm^−1^ using the attenuated total reflectance (ATR) method. For XRD analysis, Empyrean equipment with PIXcel-3D detector from PANalytical (Malvern, UK) was used in the 2 theta range from 10° to 60° with a step size of 0.02. The crystallinity index (*CrI*) of the OTP-BL and CNC was determined using the diffraction intensities of the crystalline and amorphous regions using Equation (4):

(4) $$CrI\left(\%\right)=\frac{I_{200}-I_{am}}{I_{200}}\cdot 100$$

where *I*_200_ refers to the maximum intensity of the diffraction peak of the grating (200) associated with the crystalline phase, located around 22°, and *I_am_* corresponds to the intensity of peak associated with the amorphous phase and is around 18° [38].

For the analysis of the thermal stability of the samples, thermogravimetric tests were performed using a thermogravimetric analyser TGA Q500 (TA Instruments, New Castle, DE, USA) under the following conditions: heating from 10–1000 °C, using a N_2_ flow rate of 50 mL·min^−1^. Derived thermogravimetry (DTG) curves were also obtained, and degradation temperatures were calculated using the data from the TG and DTG curves.

### 2.4. Manufacturing of Composites

Ingeo™ Biopolymer 3251D PLA (NatureWorks, Minnetonka, MN, USA) with a weight-average molecular weight (Mw) of 5.5 × 10^4^ g/mol, polydispersity index (PI) of 1.62, density of 1.24 g/cm^3^, and melt flow index of 35 g/10 min at 190 °C was selected as the polymeric matrix. Furthermore, a suitable process additive (PA), BYK P-MAX supplied by BYK-Chemie GmbH (Offenbach am Main, Germany) was also used to manufacture all the materials. BYK-MAX P 4101 is a combined processing additive based on a fatty acid ester for the use in polyolefins to optimize process control (e.g., viscosity, torque, build-up, and throughput).

To determine the influence of CNC on the PLA matrix, a wide range of composites with different percentages of reinforcement (0, 1, 3 and 5%wt.) were produced, all of them containing 1.5%wt. of PA. The maximum of 5% was chosen because according to the literature, higher addition concentrations produced an increase in CNC agglomeration. Furthermore, the incorporation of nanometer-scale particles is carried out in very small quantities (<10% by weight), obtaining a considerable improvement [17]. Table 1 shows the composition and designation of the manufactured composites.

Prior to the fabrication of the composites, moisture was removed from the PLA using a KKT 75 dryer (KOCH, Leopoldshöhe, Germany) at 60 °C for 10 h. The manufacturing process was carried out by compounding in a Rehoscam internal mixer system (Scamex, Crosne, France). PLA pellets, CNC powder, and PA powder were manually mixed for 2 min before being introduced into the internal mixer chamber. The compounding process was performed under the following conditions: temperatures of the three heating zones were maintained at 185 °C, screws rotation speed of 60 rpm, and a process time of 8 min. As a result of each batch, a homogeneous solid mass of material was finally obtained after a short cooling process outside the internal mixer. In order to obtain the material in pellet form, it was milled using a WSGM-250 milling system (J. Purchades, Madrid, Spain).

### 2.5. Characterization of Composites

The composite obtained were characterized in terms of tensile and impact strength properties. Those specimens required for the mechanical characterization were manufactured by injection moulding technology in a Victory 28 moulding machine (Engel Holding GmbH, Schwertberg, Austria). Prior to the injection moulding process, moisture was removed from the composite granulates using the KKT 75 dryer (60 °C, 10 h). The tensile properties were determined using a 10KS universal testing machine (Tinius Olsen, Redhill, UK), according to the ISO 527-2 standard [39]. A Charpy Impact Meter Izod IMPats 2281 (Metrotec, Lezo, Spain) was used to determine the impact resistance according to the ISO 179-1 standard [40].

Water absorption properties were determined using specimens of dimensions 80 × 10 × 4 mm. The method used to determine the water absorption capacity, *c*, by immersion in distilled water at 23 °C is described in ISO 62 standard [41]. The *c* values were calculated using Equation (5):

(5) $$c\left(\%\right)=\frac{m_{2}-m_{1}}{m_{1}}\times 100$$

where *m*_1_ is the initial mass of the specimen before immersion (mg), and *m*_2_ is the mass of the specimen after being subjected to the immersion process defined in the ISO 62 standard.

The fracture surface of those specimens previously used in impact tests was analysed using a Scanning Electron Microscope coupled with JSM 840 Energy Dispersive X-ray Spectroscopy (EDS) equipment (JEOL, Tokyo, Japan).

The composites were analysed in terms of structural properties by FT-IR and XRD techniques using the equipment and the data collection procedures previously described in Section 2.1.

Thermal properties of the materials were analysed by Differential Scanning Calorimetry (DSC), using a Mettler Toledo 1 Stare System (Mettler Toledo, Barcelona, Spain). The samples (each ca. 10 mg) were heated from 25 to 300 °C, then cooled to 25 °C, and finally heated again to 300 °C. All DSC curves were performed at a rate of 10 °C·min^−1^ with a N_2_ flow rate of 50 mL/min. The crystallinity (*W_c_*) of the composites was also calculated using Equation (6):

(6) $$W_{c}\left(\%\right)=\frac{∆H_{m}}{∆H_{mc}}\times \frac{1}{f}\times 100$$

where Δ*H_m_* (J/g) is the enthalpy of fusion of the analysed material, calculated as the area contained under the section of the curve corresponding to the peak of the melting process; Δ*H_mc_* (J/g) corresponds to the enthalpy of fusion of a 100% crystalline PLA (93.7 J/g) [42]; and *f* is the weight fraction of the PLA in the specific composite analysed.

## 3. Results

### 3.1. Preparation of CNCs

#### 3.1.1. Chemical Composition

**Figure 1:**
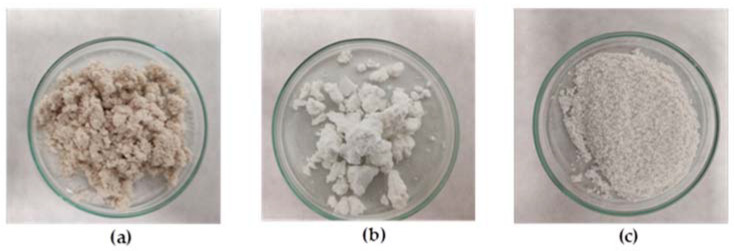
Images of samples of (**a**) OTP-BH, (**b**) OTP-BL, and (**c**) CNCs.

Figure 1 shows the appearance of OTP-BH (starting material), OTP-BL, and the CNCs obtained. The initial brown colour of the OTP-BH sample turned into pure white after the bleaching treatment with H_2_O_2_, due to the removal of non-cellulosic components such as hemicellulose and lignin [7].

The chemical composition of OTP, OTP-BH, and OTP-BL is depicted in Table 2. As shown, the hemicellulose fraction was completely removed after the treatment with H_2_O_2_, whereas the lignin content was reduced to only 3.8%wt. As a consequence, bleaching led to the obtainment of a highly purified pulp prepared for CNC synthesis in which cellulose content increased to over 86%wt. The remaining 11.20%wt. and 10.1%wt., with OTP-BH and OTP-BL, respectively, is formed by different chemical compounds such as fats, proteins, free sugars, and others, until completing the total composition of the sample, as indicated in [43]. Table 2 shows the yields of the processes carried out for the purification of the cellulose content.

#### 3.1.2. Yield of the CNC Production and Morphological Characterisation

Under our setup, the yield of the CNC synthesis was 63.92%, which is similar to that previously reported by other authors when obtaining CNC under similar conditions. For instance, Guo et al. (2020) obtained a maximum yield of 51% using 62% *v*/*v* H_2_SO_4_ when obtaining CNC from waste-tea stalks [30]. Contradictorily, Kian et al. (2020) reported lower yields (16.4–18.9%) for CNC synthesized from olive fibres that were subjected to a different process [29]. During acid treatment, the amorphous domain of cellulose fibres is attacked resulting in the generation of CNCs that may appear as agglomerates to a more or less extent. As hydrolysis proceeds, even the small crystallites can be degraded, thus leading to further isolation of singular crystals. The extent of this process and the appearance of CNCs will be therefore highly dependent not only on treatment conditions, but also on the lignocellulosic source and on the treatments performed to the fibres before synthesis of CNCs. The yield of the CNC production from the raw residue (OTP) was 13.13%.

**Figure 2:**
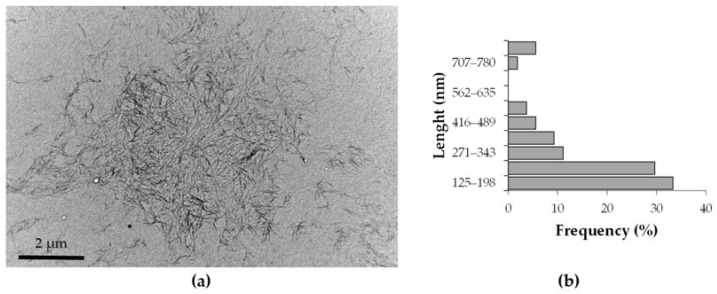
Results of the morphological characterization of CNCs: (**a**) TEM image and (**b**) particle size distribution.

Figure 2a shows the morphology of the CNCs obtained. TEM images revealed a short rod-like or whisker shape for the CNCs and some aggregates [44]. These aggregates appeared due to the strong intermolecular hydrogen bonds between the CNCs particles [7]. As illustrated in Figure 2b, an average particle size of 267 nm was obtained, with almost 90% of the particles below 500 nm in length [45]. On the other hand, the diameter of the nanocrystals obtained is between 25–50 nm. A very high L/D ratio is achieved. The size of the CNCs obtained is higher than Kian et al. (2020) reported. These authors gradually reduced the particle size of OTP CNCs from 11.35 nm width, 168.28 nm length to 6.92 nm width, 124.16 nm length varying the time of the treatments [29].

These results can be compared with the data in Table 3.

#### 3.1.3. FT-IR

**Figure 3:**
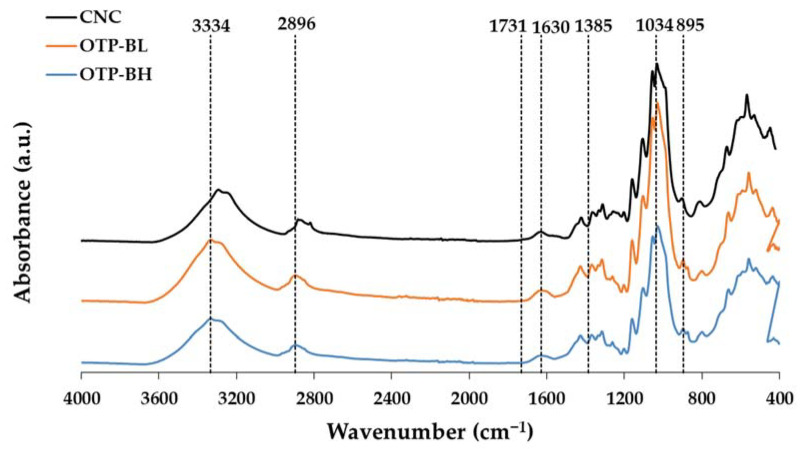
FT-IR spectra of OTP-BH, OTP-BL, and CNC.

FT-IR analysis was performed to monitor the purification of cellulose after bleaching and to study the changes in the chemical structure of sample after the CNC preparation process. Figure 3 shows the FT-IR spectra obtained for OTP-BH, OTP-BL, and CNC. In all spectra, the typical functional groups of the cellulose molecule were identified at 3334, 2896, and 1034 cm^−1^, associated with the stretching of the hydroxyl -OH group, the vibration of the C-H bond, and the C-O-C bonds (D-glucose glycosidic bonds), respectively [19,53,54]. After bleaching, a slight increase in the intensity of the peaks associated with cellulose was observed. The decrease in the peak due to the vibration of the C=O stretching associated with the acetyl and ester groups of hemicellulose and lignin, located at the wavelength of 1385 cm^−1^, stands out in the OTP-BH sample compared to OTP-BL [53]. In addition, the bands at 1731 cm^−1^ (vibration of the lignin acetyl groups) and 1649–1620 cm^−1^ (lignin aromatic ring and water absorption) were attenuated in the CNC and OTP-BL samples, with respect to the OTP-BH sample [55]. This decrease represents the removal of pectin and lignin [7]. The signal located at 895 cm^−1^, associated with β-cellulosic glycosidic bonds, was more evident in the case of OTP-BL and CNC, confirming the cellulosic type I of the CNC produced [9,55].

#### 3.1.4. XRD

**Figure 4:**
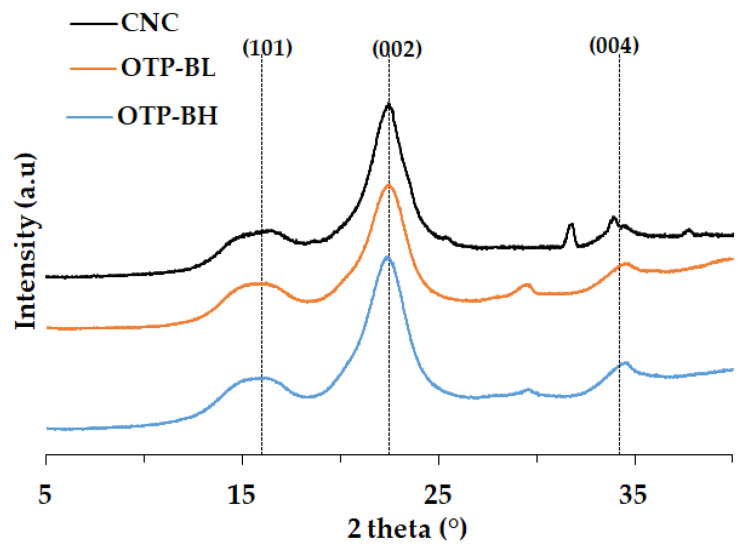
XRD patterns of OTP-BH, OTP-BL, and CNC.

Figure 4 presents the XRD patterns obtained for OTP-BH, OTP-BL, and CNC. The diffraction patterns were very similar; however, slightly sharper crystalline peaks were observed for CNC sample [7]. The highest intensity peak was observed around 22.6° due to diffraction corresponding to the (200) plane. In addition, the peak associated to the (101) plane was observed at around 16°. Both are identified with Type I cellulose [56]. As the position of these peaks was maintained after hydrolysis, it was confirmed that the treatment with sulfuric acid did not change the crystalline structure of the material [57]. The slight increase in the intensity of the peak at 22.6° for CNCs with respect to OTP-BL reflected a greater perfection of the crystalline structure [19]. In addition, the peak at 34.5°, corresponding to the crystallographic (400) plane of cellulose Type I [19,54], was more defined after CNC synthesis.

The *CrI* calculated as in Equation (4) was 70.59% for OTP-BL, whereas it increased to 74.17% for CNC. This confirms partial degradation of the amorphous domain of cellulose during the synthesis process [19,58]. Previous work obtained an increase in *CrI* from 40.92% for OTP to 71.06% for OTP fibres after a basic hydrolysis, which is in accordance with the elimination of amorphous components [36]. In line with this, Kian et al. (2020) found *CrI* of 74.8%, 79.8%, and 83.1% for cellulose nanocrystals synthesised from OTP biomass varying the conditions of the acid treatment. The trend of increasing crystallinity was in line with the decrease in crystallite size [29]. The nanocellulose *CrI* reported in this work is comparable with those for other lignocellulosic biomasses from varied sources [54,59,60] (Table 3).

#### 3.1.5. TGA

**Figure 5:**
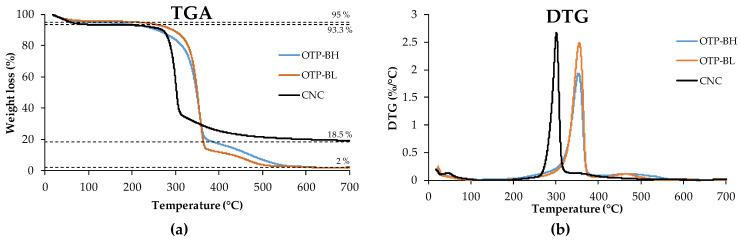
(**a**) TGA curves and (**b**) DTG curves of OTP-BH, OTP-BL, and CNC samples.

Figure 5 shows the thermal characterisation of CNC, as well as that for OTP-BH and OTP-BL samples. Although both OTP samples are depleted in hemicellulose and lignin and presented similar thermal behaviour, the thermal stability of OTP-BL was slightly increased due to the removal of non-cellulosic components after bleaching. This is reflected by the higher mass loss of OTP-BH before 300 °C and after 400 °C (Figure 5a), and by the higher intensity of the cellulose peak whose shifted from 353 °C for OTP-BH to 355 °C for OTP-BL (Figure 5b) [44].

The thermal degradation of the nanocellulose consisted of two phases. The first one, up to 100 °C, corresponded to the absorbed water, as well as to the removal of sulphate groups that adhered to the surface of the nanocellulose during the synthesis process with sulphuric acid. This event involved a weight loss of about 6.7%. The second phase corresponds to the degradation of the nanocellulose and was located between 250–350 °C. Maximum degradation of CNCs occurred at 301 °C. Generally, the thermal degradation of nanocellulose occurs at lower temperatures, compared to the raw material, due to the decrease in particle size and molecular weight [44]. In addition, after the treatment with H_2_SO_4_, there is an increase in the shorter chains and the specific surface area, generating the free chain end of the CNCs obtained by hydrolysis [9]. The degradation temperature obtained in this work is lower than that reported by Kian et al. (2020) for cellulose nanocrystals synthesised from OTP biomass varying the conditions of the acid treatment. These authors reported values between 329.1 °C and 363 °C and stated that the lower values could be due to the presence of compounds with amorphous characteristics after the treatments [29]. Fortunati et al. (2015) carried out the isolation of CNCs obtained by sulphuric acid hydrolysis of Posidonia oceanica waste, reporting a degradation temperature of 294 °C [9]. The degradation temperature reported in this work is comparable with those from other wastes [59,60] (Table 3).

The initial mass loss, corresponding to moisture and volatile matter, was greater for CNC compared to OTP-BL, likely due to its higher capability to retain water molecules. The residual mass value obtained above 600 °C also increases from 2%wt. (OTP-BL) to 18.5% (CNC). This could be attributed to the flame-retardant behaviour of crystallite structure [29].

### 3.2. Characterisation of the Manufactured Composites

#### 3.2.1. Mechanical Properties

The tensile properties obtained for PLA and the manufactured composites are shown in Table 4, indicating the values of the main parameters: maximum tensile stress (*σ_m_*), tensile stress at break (*σ_b_*), maximum tensile strain (*ε_m_*), strain at break (*ε_b_*), and Young’s modulus (*E_t_*).

As observed above, *σ_m_* decreased respect to PLA with the addition of 1 and 3%wt. CNC by 14% and 10%, respectively. Nonetheless, an increase of up to 87% was achieved by increasing the percentage of CNC to 5%wt. Small percentages of CNC do not seem to have a significant effect on the properties of the final material, which tend to be maintained or decreased slightly. The increase in resistance with higher amounts of CNC is mainly due to the larger surface area of the CNC [61]. A similar trend was observed for *σ_b_*. On the other hand, *E_t_* increased with the incorporation of CNC, reaching values 24, 56, and 58% higher for 1, 3, and 5%wt. of CNC, respectively, as compared with PLA-0CNC. Jonoobi et al. (2010) reported an improvement in *E_t_* of 24% when incorporating 5%wt. cellulose nanofibres obtained from kenaf pulp. They also observed a 21% improvement in tensile strength compared to pure PLA [8]. The increase in mechanical properties indicated that the CNCs used had good properties and that there was a good interaction between the reinforcement and the polymer matrix [61]. Good adhesion between the two phases causes efficient stress transfer, resulting in an increased strength [8,62]. As expected, the incorporation of CNCs had a negative effect on *ε_m_*, mainly due to the increased stiffness of the composite with the addition of CNC, which is a typical behaviour of reinforced/polymer composites [7,63]. The increase in stiffness may be due to the highly crystalline cellulose bonds of CNCs inhibiting the elongation of the reinforcement, affecting the behaviour of the final composite under uniaxial loading [63]. Regarding *ε_b_*, no clear trend was recorded due to the high dispersion of the results obtained. In agreement with our results, Narayanan et al. (2023) reported a decrease of 24.63% in elongation by incorporating 3 and 5%wt. of microcrystalline cellulose into a PLA matrix [31]. Jonoobi et al. (2010) fabricated PLA/CNF composites by extrusion. They reported 24 and 21% improvement in tensile modulus and tensile strength, respectively, with 5 wt% CNF to PLA. However, the elongation at break of the composite decreased significantly [8].

**Figure 6:**
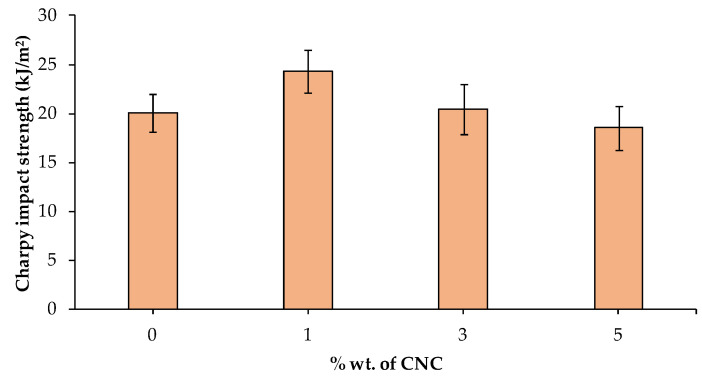
Charpy impact strength properties of PLA composites.

Figure 6 shows a comparison of the values obtained from the Charpy impact strength tests carried out on the different materials. An increase of up to 21% in impact resistance was found when adding 1%wt. of CNC. This increase may be attributed to the properties of the CNCs, mainly their high elastic modulus and aspect ratio. Compared to raw PLA, the impact resistance of the composite was practically maintained for 3%wt CNC loading, while it was reduced by up to 8% for 5%wt CNC. This can be related to the fact that the increase in CNC content leads to agglomeration phenomena, which caused impact properties to decrease [64].

#### 3.2.2. Water Absorption

**Figure 7:**
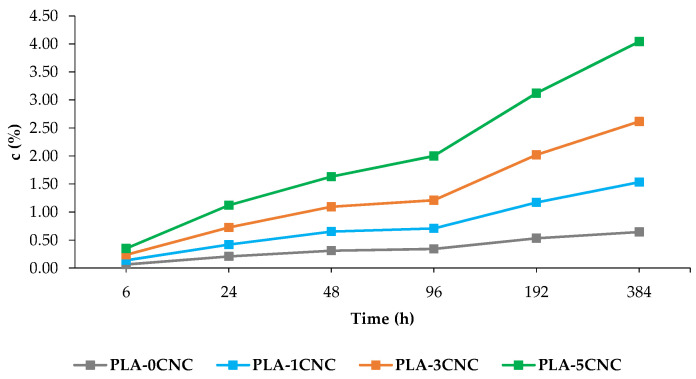
Values of *c* for PLA and composites with CNC.

Water absorption evolution of pure PLA and the composites is depicted in Figure 7. It is observed that after 384 h of immersion, the introduction of CNCs increased the *c* values from 0.643 (PLA) to 4.290% (PLA-5CNC), which means a 122% increase in the absorption capacity of the manufactured composites. CNCs are hydrophilic because of the large amount of -OH groups in their structure, which impart them higher ability for water [64]. The water absorption capacity *c* of the composites increased rapidly after 96 h [65]. The increase in the *c* values is attributed to the fact that lignocellulosic reinforcements change their particle dimensions as they incorporate water [64]. Therefore, it is logical that increasing the percentage of reinforcement resulted in a major ability of PLA composites to form interactions with water molecules. Indeed, the increase in *c* by incorporating lignocellulosic reinforcements in PLA has been reported in previous studies [4,36].

Various surface-modification techniques are currently being developed to enhance nanocellulose’s interfacial compatibility and miscibility. Some of them, i.e., esterification/acetylation, zirconium oxychloride modification could improve the hydrophobicity of the CNCs [19,64]. Besides chemical modification of the surface of nanocellulose particles, their compatibility with the continuous matrix can be potentially improved by surfactants [66].

#### 3.2.3. SEM

**Figure 8:**
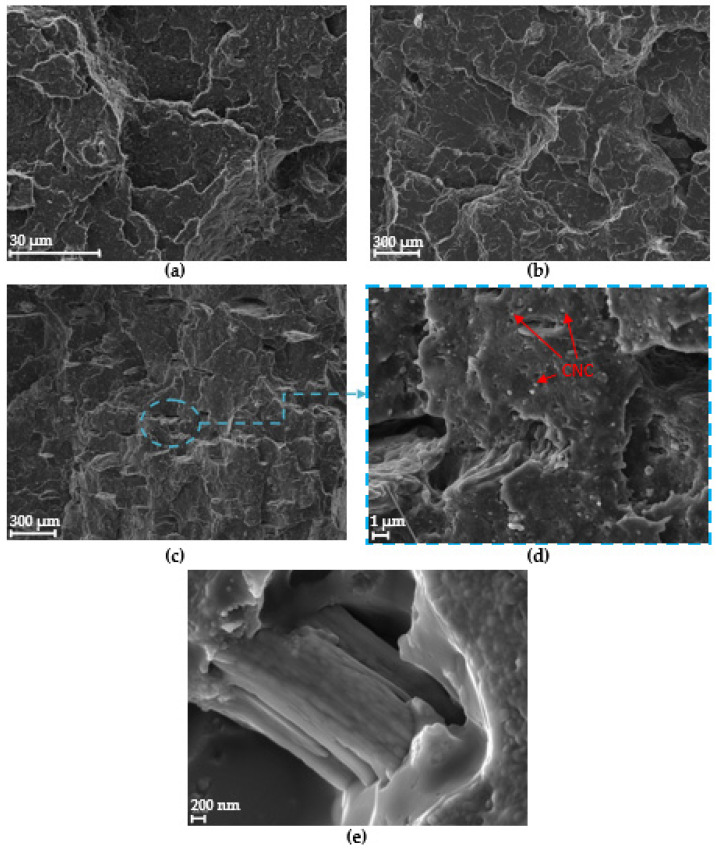
SEM images for (**a**) PLA-0CNC, (**b**) PLA-3CNC (**c**) PLA-5CNC, (**d**) PLA-5CNC detail, and (**e**) CNC detail.

For the study of the fracture surface, suitable SEM images were obtained with the aim of corroborating the presence of CNCs. Microscopic aspects of the fracture surface of composite samples can provide a good explanation for the improved mechanical properties. Figure 8a shows the fracture surface of the raw PLA sample in the form of layers. The surface of the composites (Figure 8b,c), did not show significant differences with raw PLA [8]. A homogeneous distribution of the CNCs into the polymer matrix was observed in all the composite samples, which is very positive in terms of mechanical properties because when good dispersion of loads occurs, the neighbouring CNCs prevent the propagation of cracks [67]. According to Figure 8d, it seems to be a good interaction between the CNCs and the PLA matrix. This good interaction is responsible for the improvement in the mechanical properties reported above due to the effective stress transfer occurred at optimum loading (5 wt.% of CNC) [67]. Figure 8e shows a detail of CNCs agglomerated in bundles.

#### 3.2.4. FT-IR

**Figure 9:**
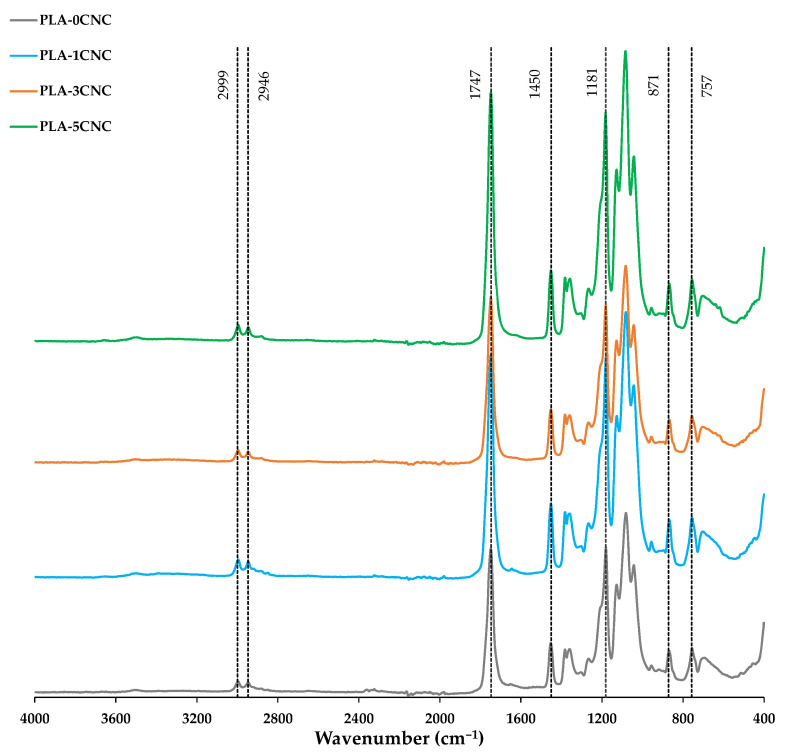
FT-IR spectra for PLA and composites.

The chemical nature of pure PLA and composites was explored by FT-IR analysis. The resulting spectra are shown in Figure 9, where the characteristic peaks of PLA can be easily appreciated. The -OH bending is located in the 3330–3600 cm^−1^ range [68]. The characteristic band with high absorption peak in the carbonyl region (1747 cm^−1^) is attributed to the ester bond in the PLA backbone [31,69]. The peaks between 2946 and 2999 cm^−1^ are due to asymmetric C-H stretching [70] and those present at 1181 and 1130 cm^−1^ correspond to C-O-C and C-C stretching vibration, respectively [69]. The peaks at 1450 cm^−1^ are attributed to the deformation of the CH single bond into CH_3_, assigned to lactic acid, and the peak corresponding to the C-C single bond present at 871 and 757 cm^−1^ [4,31,64].

Due to the small level of CNC loading in the matrix, the spectra of the composites did not differ significantly to that of neat PLA, producing a superposition of the PLA and CNC spectra [67]. Furthermore, after adding CNCs, the position of all those peaks corresponding to PLA were maintained, indicating that the addition of CNCs did not change the PLA structure; however, an increase in the band associated with the -OH bond attributed to cellulose was observed around 3300 cm^−1^ [68,69]. The lack of any additional peak suggested that the interaction between the reinforcement and the matrix occurred in physical and not chemical terms, and that the CNC loading was not sufficiently high to observe significant differences [65].

#### 3.2.5. XRD

**Figure 10:**
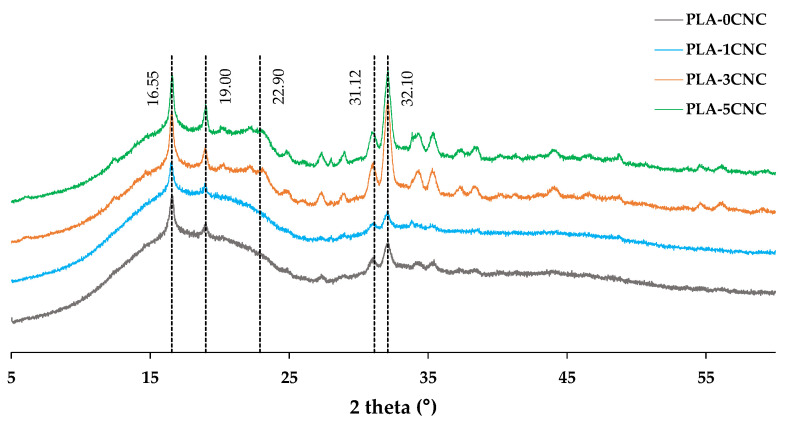
XRD patterns for PLA and composites.

The crystalline characteristics of the CNC-reinforced PLA composites were examined with XRD patterns (Figure 10). A hump between 15–23° and a valley centred at 28° were identified in all the patterns, which is indicative of a semi-crystalline structure [1,31]. This structure is typical of PLA [71]. Typical sharp peaks at 16.55°, 19.00°, and 32.10° were also identified [31]. The diffraction peak at 19.00° corresponds to the crystallisation phase of PLA α’ [4]. On the other hand, the diffraction plane appearing around 22.90° was attributed to the (200) plane of cellulose. Therefore, the intensity of this peak became more pronounced, indicating that this signal involves the PLA and the CNC [70,71].

#### 3.2.6. DSC

**Figure 11:**
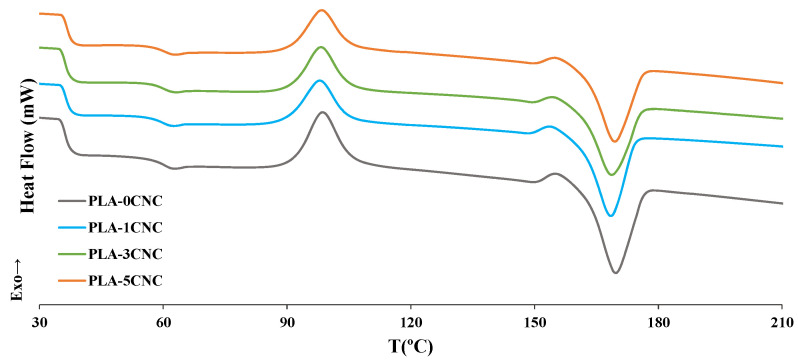
DSC curves of PLA and composites.

Figure 11 shows the curves obtained from the second heating of the DSC tests. Compared to PLA, the heating curves of composites did not change significantly, indicating that the reinforcement did not affect the crystallisation process of PLA [67].

The main thermal parameters determined from the DSC curves, namely glass transition temperature (*T_g_*), crystallization temperature (*T_c_*), melting temperature (*T_m_*), heat of fusion (Δ*H_f_*), and percentage of crystallinity (*W_c_*), are summarized in Table 5.

No significant differences were found when CNCs were added up to 5%wt. with respect to pure PLA. This can be attributed to the negligible influence of the CNC-PLA interactions on the mobility of the polymer chains during the glass transition [9,61]. A small exothermic phase can be observed around 150 °C, corresponding to the crystalline transformation from the α’ form to the α form of PLA [48]. Robles et al. (2015) reported similar results to those obtained in this study, indicating that the addition of CNC and CNF to the PLA matrix did not significantly change *T_g_*, *T_c_*, and *T_m_* [50]. Compared to raw PLA, the crystallinity of the composite was maintained or even slightly decreased, with the exception of PLA-1CNC. This decrease does not follow the trend recorded for other composites, but it represents a very small variation (less than 2%). According to Sung et al. (2017), CNCs may act as nucleation agents into a PLA matrix when good particle dispersion is achieved [7]. Therefore, the slight decrease in *W_c_* and Δ*H_c_* reported in our work may indicate a certain level of CNC agglomeration with CNC concentration higher than 1%wt., thus preventing them from acting as nucleation agents [31,67].

## 4. Conclusions

CNCs were synthesised by sulfuric acid hydrolysis from OTP residues. The initial purification and bleaching processes led to a cellulose content of up to 86%wt., with no hemicellulose detected and a significant decrease in lignin content (from 5%wt. to 3.8%wt.). The final CNCs obtained showed an average length of 267 nm, high thermal stability (degradation temperature above 300 °C), and crystallinity of 74%. PLA-based composites were successfully manufactured, incorporating up to 5%wt. of the previously synthesised CNCs as reinforcement into the PLA polymer matrix. The incorporation of CNCs had a significant influence on the material properties. *E_t_* and *σ_m_* increased by 58% and 87%, respectively, when 5%wt. CNC was added, mainly due to a good interaction between the reinforcement and the matrix, favouring the transmission of stresses. Charpy impact strength improved by 21% with 1%wt. of CNC loading. SEM images showed a homogeneous distribution of the reinforcement into the polymer matrix. In general, thermal properties of the composites were maintained. However, a slightly decrease in crystallinity was reported when adding the highest percentages of reinforcement (3 and 5%wt.), which was attributed to the agglomeration of the CNCs in bundles, which prevented them from acting as nucleation agents. All the results obtained suggest that the manufactured composites have a high potential as a new biomaterial. These composites were manufactured using industrially common manufacturing methods (melt processing and injection moulding), showing these composites as a possible replacement for conventional plastics.

## Figures and Tables

**Table 1:** Composition of manufactured biocomposites.

Composite	PLA (%wt.)	CNC (%wt.)	PA (%wt.)
PLA-0CNC	98.5	0	1.5
PLA-1CNC	97.5	1	1.5
PLA-3CNC	95.5	3	1.5
PLA-5CNC	93.5	5	1.5

**Table 2:** OTP-BH and OTP-BL composition (%wt.).

	Moisture (%wt.)	Ash (%wt.)	Cellulose (%wt.)	Hemicellulose (%wt.)	Lignin (%wt.)	Yield (%)
OTP *	7.00	0.18	31.50	21.60	24.80	-
OTP-BH *	3.98	0.03	83.30	0.50	5.00	22.9
OTP-BL	5.62	0.00	86.10	Not detected	3.80	89.7

* [36].

**Table 3:** Main characteristics of CNC from wastes.

Waste	Length (nm)	Diameter (nm)	Crystallinity (%)	Degradation Temperature (°C)	Reference
Sunflower seed	450.0 ± 50.0	50.0 ± 15.0	82.0	200	[46]
Cotton	177.0	12.0	90.5	-	[47]
Corn	287.3 ± 75.5	4.9 ± 1.3	79.8	200	[48]
Corn stover	356.3 ± 98.0	7.0 ± 1.9	55.0	-	[49]
Sugarcane baggasse	37.0–220.0	18.0–32.0	-	-	[50]
	250.0–480.0	20.0–60.0	72.5	236	[45]
Plum seed shells	100.0–800.0	14.0	54.0	196	[51]
Rice Straw	116.6–166.0	3.9–6.7	90.7	-	[52]

**Table 4:** Tensile properties of PLA and composites.

Composite	*σ_m_* (MPa)	*σ_b_* (MPa)	*ε_m_* (%)	*ε_b_* (%)	*E_t_* (MPa)
PLA-0CNC	45.42 ± 4.00	35.52 ± 4.65	4.84 ± 0.38	9.75 ± 1.90	3033.25 ± 174.26
PLA-1CNC	39.10 ± 2.97	31.17 ± 2.82	3.66 ± 0.28	10.52 ± 1.18	3770.15 ± 244.96
PLA-3CNC	41.04 ± 1.14	34.14 ± 1.18	3.48 ± 0.27	5.44 ± 1.03	4734.97 ± 229.29
PLA-5CNC	85.07 ± 5.19	63.97 ± 6.78	3.75 ± 0.34	8.23 ± 2.07	4812.18 ± 282.70

**Table 5:** Thermal properties of PLA and composites.

Composite	*T_g_* (°C)	*T_c_* (°C)	*T_m_* (°C)	Δ*H_c_* (J/g)	Δ*H_m_* (J/g)	*W_c_* (%)
PLA-0CNC	62.51	98.59	170.60	25.13	36.18	39.20
PLA-1CNC	62.31	97.94	169.13	22.35	37.35	40.89
PLA-3CNC	62.65	98.28	169.42	22.25	31.99	35.75
PLA-5CNC	62.81	98.67	170.27	20.92	32.85	37.50

## Data Availability

The data presented in this study are available on request from the corresponding author.
